# Improvement of ACK1-targeted therapy efficacy in lung adenocarcinoma using chloroquine or bafilomycin A1

**DOI:** 10.1186/s10020-023-00602-z

**Published:** 2023-01-16

**Authors:** Jinhong Zhu, Kui Cao, Meng Zhao, Keru Ma, Xiangyu Jiang, Yuwen Bai, Xiaodong Ling, Jianqun Ma

**Affiliations:** 1 Department of Clinical Laboratory, Biobank, Harbin Medical University Cancer Hospital, 150 Haping Road, Harbin, 150040 Heilongjiang China; 2 Department of Thoracic Surgery, Harbin Medical University Cancer Hospital, 150 Haping Road, Harbin, 150040 Heilongjiang China

**Keywords:** ACK1, LUAD, Chloroquine, Bafilomycin A1, AMPK, Prognosis

## Abstract

**Background:**

Activated Cdc42-associated kinase 1 (ACK1) is a promising druggable target for cancer, but its inhibitors only showed moderate effects in clinical trials. The study aimed to investigate the underlying mechanisms and improve the antitumor efficacy of ACK1 inhibitors.

**Methods:**

RNA-seq was performed to determine the downstream pathways of ACK. Using Lasso Cox regression analysis, we built a risk signature with ACK1-related autophagy genes in the lung adenocarcinoma (LUAD) patients from The Cancer Genome Atlas (TCGA) project. The performance of the signature in predicting the tumor immune environment and response to immunotherapy and chemotherapy were assessed in LUAD. CCK8, mRFP-GFP-LC3 assay, western blot, colony formation, wound healing, and transwell migration assays were conducted to evaluate the effects of the ACK1 inhibitor on lung cancer cells. A subcutaneous NSCLC xenograft model was used for in vivo study.

**Results:**

RNA-seq revealed the regulatory role of ACK1 in autophagy. Furthermore, the risk signature separated LUAD patients into low- and high-risk groups with significantly different prognoses. The two groups displayed different tumor immune environments regarding 28 immune cell subsets. The low-risk groups showed high immune scores, high CTLA4 expression levels, high immunophenoscore, and low DNA mismatch repair capacity, suggesting a better response to immunotherapy. This signature also predicted sensitivity to commonly used chemotherapy and targeted drugs. In vitro, the ACK1 inhibitors (AIM-100 and Dasatinib) appeared to trigger adaptive autophagy-like response to protect lung cancer cells from apoptosis and activated the AMPK/mTOR signaling pathway, partially explaining its moderate antitumor efficacy. However, blocking lysosomal degradation with chloroquine/Bafilamycine A1 or inhibiting AMPK signaling with compound C/shPRKAA1 enhanced the ACK1 inhibitor’s cytotoxic effects on lung cancer cells. The efficacy of the combined therapy was also verified using a mouse xenograft model.

**Conclusions:**

The resulting signature from ACK1-related autophagy genes robustly predicted survival and drug sensitivity in LUAD. The lysosomal degradation inhibition improved the therapeutic effects of the ACK1 inhibitor, suggesting a potential role for autophagy in therapy evasion.

**Supplementary Information:**

The online version contains supplementary material available at 10.1186/s10020-023-00602-z.

## Introduction

Lung cancer remains top-ranked in incidence and mortality globally (Bray et al. 2018), 85% of which is non-small cell lung cancer (NSCLC). Most patients are not diagnosed until they develop locally advanced or widely metastatic tumors (Herbst et al. 2018). As a result, the 5-year survival of lung cancer is as low as 15%, despite dramatic progress in multimodality therapy (Ashrafizadeh et al. 2020; Herbst et al. 2018). Aberrant activities of many fundamental signaling pathways are responsible for the uncontrol growth and metastasis of NSCLC, including the AMPK, KRAS/RAF/MEK, PI3K/AKT/mTOR, and JAK-STAT pathways (Ashrafizadeh et al. 2021a, b; Herbst et al. 2018; Rotow and Bivona 2017). In the era of precision medicine, targeted therapies have caused a revolutionary improvement in cancer management by inhibiting activating genetic alterations in the epidermal growth factor receptor (EGFR), anaplastic lymphoma kinase (ALK), and ROS1 proto-oncogene receptor tyrosine kinase (ROS1). However, advanced-stage NSCLC remains rarely cured because of therapeutic resistance (Ashrafizadeh et al. 2021a, b; Bivona and Doebele 2016; Rotow and Bivona 2017). Therefore, it is urgent to develop more potent targeted therapies. Equally important is to identify the targetable resistance mechanisms of existent drugs and develop a combinational therapy strategy to overcome the resistance.

Activated Cdc42-associated kinase 1 (ACK1), also known as a non-receptor tyrosine kinase 2 (TNK2), is a ubiquitously existing non-receptor tyrosine kinase (NRTK) (Wang et al. 2021). *ACK1* gene is frequently amplified or mutated in a broad spectrum of human cancer, along with aberrantly activated ACK1 signaling (Mahajan et al. 2015). This atypical receptor tyrosine kinase converges and transduces signals from multiple ligand-activated RTKs (e.g., EGFR, HER2, and PDGFR) to downstream effectors (Mahajan et al. 2015). Therefore, ACK1 has been considered a promising therapeutic target for multiple cancers, including colorectal cancer (Qi and Ding 2018), breast cancer (Mahajan et al. 2015), hepatocellular carcinoma (HCC) (Lei et al. 2015), head and neck squamous carcinoma (Peng et al. 2022), and NSCLC (Gu et al. 2020; Hu et al. 2016; Tan et al. 2014; Zhu et al. 2020; Zhu et al. 2021a, b). One of the mechanisms underpinning tyrosine kinase inhibitor (TKI) resistance is that drugs often unintentionally activate signaling molecules (e.g., MET, BRAF, PIK3CA, AXL, and Src family kinases). The subsequent activation of these parallel signaling pathways can adequately support cell survival and growth and thereby cause tumor cells to evade TKI-mediated tumor cell killing (Zhang et al. 2020; Zhu et al. 2021a, b). Because ACK1 integrates signals from several RTKs, its inhibitors may restrict the compensatory activation of the signaling pathways mentioned above and minimize drug resistance. Therefore, ACK1 inhibitors may be a better therapeutic strategy for cancer treatment. Many ACK1 inhibitors have been identified, such as Dasatinib, Sunitinib, and (*R*)-9b (Ghildiyal et al. 2022; Wang et al. 2021). However, clinical responses of Dasatinib were dissatisfying (Kelley et al. 2017; Kruser et al. 2011). Therefore, it is warranted to investigate the potential resistance mechanism of ACK1 inhibitors.

Autophagy is a programmed catabolic process assisting cells to survive stresses, such as hypoxia, starvation, or cytotoxic drugs (Zhao et al. 2021). Autophagosomes coupled with lysosomes to eradicate protein aggregates and damaged detrimental organelles (e.g., mitochondria). Autophagy is a double-edged sword in cancer development (Levy et al. 2017; White and DiPaola 2009). Autophagy may prevent tumorigenesis-driving genome damage by removing damaged proteins and organelles (White and DiPaola 2009). Some evidence demonstrated that impaired autophagy, arising from a deficiency in beclin 1 or the autophagy gene Atg5, is tumor-promoting. In contrast, autophagy can also disintegrate cellular contents to fuel tumor cell growth and facilitate tumor cells to survive metabolic stress, thereby promoting tumorigenesis (White and DiPaola 2009; Xia et al. 2021). Several signaling pathways have been found to regulate autophagic activity, involving PI3K/AKT/mTOR, AMPK/CaMKK, RAS, p53, and JAK-STAT pathways (Galluzzi et al. 2017; Levy et al. 2017). Over the past years, accumulating evidence illustrated that autophagy is a crucial drug resistance mechanism in anticancer therapy (Holohan et al. 2013; Kwon et al. 2019). Many anticancer therapies stimulate autophagic pathways, including chemotherapy and targeted therapies. Autophagy inhibitors, chloroquine or hydroxychloroquine, combined with several anticancer drugs, could elevate cytotoxicity in preclinical models (Chang and Zou 2020; Ishaq et al. 2020). Intriguingly, the effect of autophagy on cells is context-dependent. For instance, arsenic trioxide (ATO) inhibits cancer cell growth by triggering autophagic cell death (Agrawal et al. 2022). To date, it remains unknown whether ACK1 regulates cellular autophagic activity or whether ACK1 inhibitors stimulate self-defense autophagy to resist their therapeutic effect.

In this study, we developed a predicting signature of ACK1-related autophagy genes. This signature potently predicted prognosis, chemotherapy response, and drug sensitivity in LUAD. Moreover, by performing in vitro and in vivo experiments, we found that inhibiting AMPK or lysosomal degradation with Chloroquine (CQ)/ Bafilomycin A1 (BafA1) boosted the cytotoxic effects of ACK1 inhibitors on lung cancer cells.

## Methods and materials

### RNA-sequencing

Details of the silencing of ACK1 in A549 cells by lentivirus-medicated shRNA and RNA-sequencing were provided elsewhere (Zhu et al. 2021a, b).

### Bioinformatics analysis

#### Construction of a multiple gene signature with ACK1-associated autophagy genes

We retrieved 20,198 ACK1-related genes (*P* < 0.05) from the TCGA-LUAD cohort and 232 autophagy genes Human Autophagy Database (HADb, http://autophagy.lu/clustering/index.html). Totally, 149 autophagy genes overlapping ACK1-associated genes were defined as ACK1-related autophagy genes. We further screened for genes significantly associated with overall survival in the TCGA-LUAD cohort using the univariate Cox regression analysis. The significant genes were entered into the least absolute shrinkage and selection operator (LASSO) Cox regression algorithm to establish an optimal risk model with ACK1-related autophagy genes as published previously (Cao et al. 2021a, b; Liu et al. 2020, 2019).

Next, using a risk score formula linearizing the expression levels of the gene signature in LUAD patients, we quantitated the risk for unfavorable survival for each patient (Liu et al. 2019; Zhu et al. 2021a, b). Furthermore, we built a Cox-based nomogram to test the ability of the risk score to predict personal OS, with a concordance index (C-index) to measure its discriminative ability (Iasonos et al. 2008). Nomogram-predicated probability was compared with the observed outcome by plotting the calibration curve. Decision curve analysis (DCA) was used to assess the clinical net benefit of the risk score compared to ACK1 and TNM stage (Vickers et al. 2008; Vickers and Elkin 2006).

#### Evaluation of tumor immune environment and drug sensitivity

We first estimated the association of risk score with immune checkpoint genes or DNA mismatch repair genes with the R package ggstatsplot in the TCGA-LUAD cohort because they were considered as a predictive biomarker for immunotherapy (Chan et al. 2019; Le et al. 2017; Luchini et al. 2019; Rizvi et al. 2018). Based on the metagene methodology, we used the single-sample Gene Set Enrichment Analysis (ssGSEA) algorithm to calculate the fraction of 28 immune cell subpopulations in LUAD (Charoentong et al. 2017). We also downloaded the immunophenoscore (IPS) dataset for LUAD patients from The Cancer Immunome Atlas (TCIA, https://tcia.at/home). The IPS was derived by comprehensively integrating four crucial tumor immunogenicity determinants, that is, major histocompatibility complex (MHC) molecules (antigen processing), effector cells, immunosuppressive cells, as well as checkpoints and immunomodulators (Charoentong et al. 2017).

Moreover, to predict other drug sensitivity, an R package named pRRophetic was generated (Geeleher et al. 2014a, b) with reference to gene expression microarray data of near 700 cell lines before and after the administration of 138 drugs from the Cancer Genome Project (CGP). This R package (https://github.com/paulgeeleher/pRRophetic) permitted us to predict clinical drug sensitivity by analyzing gene expression profiles of tumors. By applying this methodology, we calculated half inhibitory concentrations (IC50) of standard anticancer drugs for both high- and low-risk groups.

### Cell culture

We obtained human lung bronchial epithelial (BEAS-2B, cat# CBP60577) from COBIOER Biosciences CO., LTD (Nanjing, China) and a number of lung cancer cells from the Cell Bank of the Chinese Academy of Science (Shanghai, China), including A549 (cat# SCSP-503), PC-9 (cat# SCSP-5085), HCC827 (cat# SCSP-538), NCI-H460 (cat# SCSP-584), NCI-H1299 (cat# SCSP-589), NCI-H1915 (cat# SCSP-597), and H1650 (cat# SCSP-592). Cells were maintained in the recommended medium, DEME medium for Beas-2B, or RPMI 1640 for the rest of the cell lines (Procell, China) with the addition of 10% fetal bovine serum (Hyclone, Life Sciences, Shanghai, China), penicillin G (100 U/ml, Beyotime, China), streptomycin (100 μg/ml, Corning, China) in a humidified incubator with 5% CO_2_, at 37 °C.

### Lentivirus infection

Lentivirus encoding shRNA targeting ACK1/TNK2 and a negative control shRNA were purchased from GeneChem (Shanghai, China). Sequence targeting ACK1 (RNAi, tgCTTCCT CTTCCACCCAATT) were inserted in pLVshRNA-puro. A pLVX-Puro vector carrying the coding DNA sequence (CDS) region of ACK1/TNK2 was obtained from the same company and used to overexpress ACK1 in the lung cancer cells. Lentivirus infection was conducted on cells while they reached 80% confluency, with a multiplicity of infection (MOI) of 50. The shACK1 cells, ACK1 overexpression cells, and respective control cells were passaged in a culture medium supplemented with puromycine to establish stable cell lines. Total RNA was extracted to detect ACK1 mRNA expression levels using TRIzol reagent (Invitrogen, Thermo Fisher Scientific, USA). ACK1 transcripts were amplified using real-time PCR with GAPDH as an internal control. The primer information was referred to a previous publication.

### Plasmids, transfection, and RNA interference

Plasmid (hU6-MCS-Ubiquitin-EGFP-IRES-puromycin) overexpressing PRKAA1 shRNA and controls were obtained from Shanghai Genechem Co., Ltd. (China). We used the shRNA sequence CATAAAGTAGCTGTGAAGATA to knock down the PRKAA1 gene. While reaching 80% confluence, cells in 6-well plates were transfected with control plasmids or plasmid encoding shRNAs targeting PRKAA1 (50 nM) using jetPRIME® transfection reagent (Polyplus Transfection Inc. New York, NY, USA). Cells were maintained in a culture medium for 24 h before being used for experiments.

### Materials and reagents

AIM-100, chloroquine (CQ), and bafilomycin A1 (BafA1) were purchased from MedChemExpress (MCE, Princeton, NJ, USA). Antibodies purchased from Cell Signaling Technology (Danvers, MA, USA) were as follows: anti-ACK1, anti-total AMPK and anti-phosphorylated-AMPKα1 (Ser485), anti-total and anti-phosphorylated-mTOR (Ser2448), anti-Agt5, anti-beclin 1, anti-LC3, and anti-p62 antibodies. Antibody against phosphorylated-ACK1 (Y284) was obtained from Abcam Inc. (Cambridge, MA, USA).

### Apoptosis analysis

Apoptosis was detected with an Annexin-V APC detection kit (eBioscience, USA). Briefly, cells subjected to different treatments were harvested and incubated with anti-Annexin V antibody labeled with APC and PI for 10 min in the dark, following the protocol provided by the manufacturer. Apoptotic cells were quantified using FACS Calibur flow cytometry.

### Western blot analysis

Cells were grown in the 75 mm flask and treated with different concentrations of drugs. We collected and disintegrated cells in RIPA buffer at indicated times. Whole-cell extracts were prepared and separated in SDS-PAGE and blotted onto a polyvinylidene difluoride membrane. Blots were visualized using Immobilon Western Chemiluminescence HRP substrate (Millipore, Billerica, MA). If needed, blots were washed off using a stripping buffer, followed by reprobation with different primary antibodies.

### Cell viability assay

The cancer cell suspension was added to 96-well plates at 1000 to 5000 cells/wells. After attachment, cells were treated with various concentrations of AIM-100 for 72 h. Cell viability was determined using Dojindo cell counting kit-8 (CCK-8, GlpBio, USA) at 24, 48, and 72 h, following the manufacturer’s instructions.

### Colony formation assay

Cells were plated in a 12-well plate at a density of 800 cells per well. Cells grew for ten days in a 37 °C incubator until small cell colonies were observed with the naked eye. Then, cells were fixed with 4% paraformaldehyde for 20 min, followed by staining with 0.2% crystal violet at room temperature. Image J was used to quantify the relative density of colonies with different treatments.

### Wound healing assay

A549 cells were cultured in the 6-well plates. While cells reached 95% confluence, vertical scratches were created on monolayers. And afterward, a serum-free medium was used to maintain cells. Images were taken, and gaps in the wounds were measured at 0 h and 18 h.

### Migration assay

Migration assays were carried out using Transwell chambers (8 µm; Corning, Tewksbury, MA, USA). Briefly, cells were harvested, washed, and resuspended. Cell suspension with serum-free DMEM was added to the upper wells of the chambers at a density of 5 × 10^4^ cells/well, whereas the lower wells of the chambers contained DMEM supplemented with 10% FBS serving as a chemoattractant. The transwell chambers were maintained in a 37 °C incubator for 18 h to allow cells to migrate to the lower surface of the filter. Migrated cells were fixed with 4% paraformaldehyde, visualized with 0.1% crystal violet, and numerated under a microscope.

### Monitoring autophagosome formation

Various methods have been developed to measure autophagy, including the long-lived protein degradation assay, the lactate dehydrogenase sequestration assay, and the mRFP-GFP-LC3B fusion protein assay (Klionsky et al. 2021, 2016; Luhr et al. 2018a, b; Luhr et al. 2018a, b). Adenoviral vectors expressing mRFP-GFP-LC3 fusion protein and empty vectors were obtained from HanBio (Shanghai, China). A549 cells infected with adenoviral vectors were cultured for 24 h to allow the expression of mRFP-GFP-LC3B fusion protein. And then, cells were treated with AIM-100 (20 μm) or Dasatinib (20 μm) for an additional 12. Cells treated with DMSO served as control. After fixation with 4% paraformaldehyde (PFA) for 15 min, images of cells were taken using NIKON-TS2 fluorescence microscopy (Nikon Instruments Inc., Japan) with NIS-Elements F imaging software). The mRFP-GFP-LC3 fusion proteins were diffused in the cytoplasm of cells; therefore, both fluorophores fluoresce were very weak. Upon the stimulation, mRFP-GFP-LC3 fusion proteins were recruited to the membrane of autophagosomes. As a result, autophagosomes could be visualized as yellow fluoresce puncta. When an autophagosome fused with a lysosome, only red puncta can be observed because GFP signals were quenched in the lower pH environment of the autolysosome.

### In vivo tumorigenesis study

The Institutional Review Board of Harbin Medical University Cancer Hospital approved the animal study protocol. Female BALB/c nude mice at 4–5 weeks of age were purchased from Charles River (Beijing Vital River Laboratory Animal Technology Co., Ltd., China). Mice were kept under specific pathogen-free conditions.

Every mouse received a dose of 1 × 10^6^ A549 cells through subcutaneous injection. Mice were randomized into four groups ten days post-injection: Group 1: 0.1% DMSO as vehicle control; Group 2: CQ (30 mg/kg) via intraperitoneal injection every other day; Group 3: Dasatinib (30 mg/kg) administrated intragastrically; Group 4: the combination of CQ (30 mg/kg) and Dasatinib (20 mg/kg). The dosages of ACK1 inhibitor, Dasatinib (20 mg/kg), and autophagy inhibitor, CQ, were determined based on previous publications (Wang et al. 2018; Zhang et al. 2020), and both drugs were administrated every other day for four weeks. Tumor sizes were measured and recorded regularly. Mice were euthanized, and tumors were harvested and weighed at the end.

### Statistics

The statistical analysis was performed using SPSS version 22 (SPSS, Inc., Chicago, IL, USA) and R version 4.0.3 (https://www.r-project.org/). We integrated the differences between two groups using the Student’s *t*-test, and the differences among three groups or more were tested with one-way ANOVA. If a significant result was found in the latter test, Tukey’s multiple comparisons tests were performed to determine which two groups the significant difference existed. Kaplan–Meier survival curves of OS were plotted for high- and low-risk groups. Receiver operating characteristic (ROC) curves were used to determine the prognostic accuracy of risk factors. Both univariate and multivariate Cox proportional hazards regression analyses were conducted to assess the association of the combined score and clinicopathological characteristics with overall survival. *P* < 0.05 was considered to be significant.

## Results

### Association of ACK1 with autophagy and generation of a prognostic signature with ACK1-related autophagy genes

**Fig. 1:**
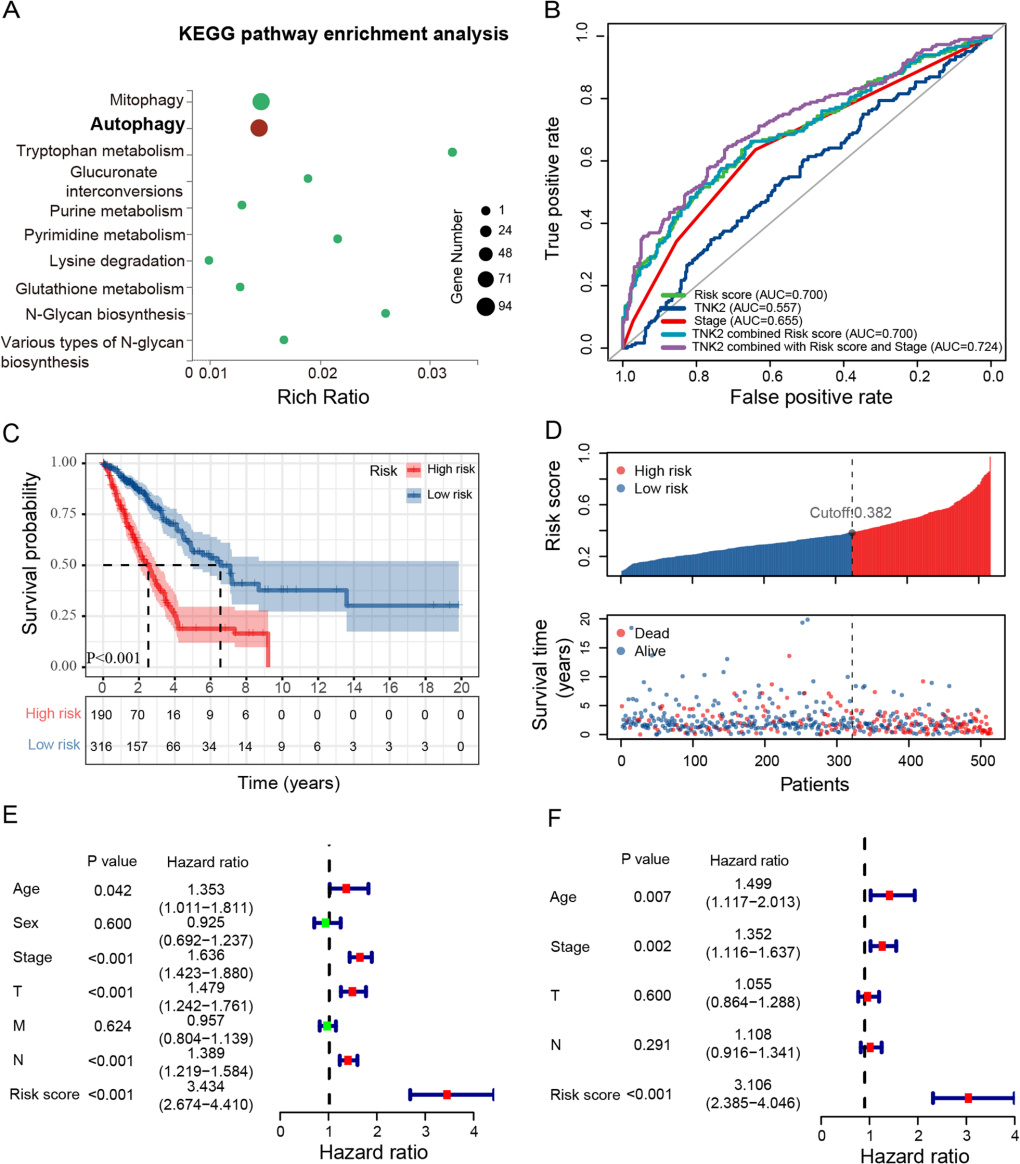
The association of ACK1 with autophagy and the prognostic accuracy of the risk signature of ACK1-related autophagy genes in the TCGA-LUAD cohort. **A** RNA-seq was performed to examine RNA expression profilings in shControl and shACK1 A549 cells. Differentially expressed genes were enriched in autophagy. The LASSO regression model was used to identify ACK1-correlated autophagy genes contributing most to prognosis and built the best risk signature in the TCGA-LUAD cohort. **B** The 5-year ROC curves with the risk score show a higher AUC value than other single risk factors. **C** The Kaplan–Meier survival curves indicated overall survival for low- and high-risk LUAD patients defined by the risk signature. **D** The relation between the risk score and survival status of the TCGA-LUAD patients. Forest plots of univariate **E** and multivariate **F** Cox regression analyses

A549 cells were used for the RNA-Seq screening. Differentially expressed genes, retrieved from gene expression profiles of control and ACK1 knockdown cells, were used to interrogate ACK1-regulated signaling pathways and cellular events. A total of 1,076 differentially expressed genes (DEGs) were identified in cells with ACK1 knockdown compared to controls (Zhu et al. 2021a, b). Interestingly, Gene Ontology (GO) and Kyoto encyclopedia of genes and genomes (KEGG) enrichment analysis revealed that DEGs were enriched in autophagy (Fig. 1A), a cellular process related to drug resistance in various cancer. The association of ACK1 with survival in NSCLC is controversial (Hu et al. 2016; Tan et al. 2014). A single biomarker cannot fully recapitulate the distinct molecular heterogeneity of cancer. Therefore, ACK1 alone may not adequately predicate prognosis in NSCLC robustly. Increasing publications demonstrated that integrating biomarker genes and their functional relevance to carcinogenesis yielded gene signatures greatly predictive of cancer outcomes (Sheng et al. 2020; Zhu et al. 2020; Zhu et al. 2021a, b). Because of the close linkage between ACK1 and autophagy, we are motivated to ameliorate the prognostic accuracy of ACK1 by considering ACK1’s role in autophagy. The relevance of ACK1 to autophagy may unveil its implications for cancer outcomes and drug resistance. We identified 149 ACK1-associated autophagy genes, among which univariate Cox analysis was conducted to identify 32 genes significantly associated with survival in the TCGA-LUAD cohort. Using the LASSO algorithm, we generated an optimal signature consisting of 23 ACK1-related autophagy genes (Additional file 1: Fig. S1A, B). A risk score formula was adopted to quantify patient risk for unfavorable survival. Every patient received a risk score based on each gene’s expression level and risk coefficient in the signature (Table 1). While plotting the ROC curves with indicated parameters, the risk score reached the highest AUC value of 0.700, and TNK2 combined with the TNM stage and risk score achieved a maximum AUC value of 0.724 (Fig. 1B). We next plotted time-dependent ROC for the risk score against OS. The risk score with the highest Youden index in 3-year ROC was used as the best cutoff value to divide patients into high- and low-risk groups (Additional file 1: Fig. S1C). Kaplan–Meier survival analyses indicated that life spans of LUAD patients with high risk were significantly shorter than those of low-risk patients (Fig. 1C). High-risk LUAD patients showed a significantly higher frequency of cancer death than their low-risk counterparts (Fig. 1D). Moreover, univariate and multivariate Cox regression analyses revealed that risk score is an independent prognostic LUAD (Fig. 1E, F).

**Table 1:** List of genes included in the risk signature

Gene	Information	Coefficient
APOL1	Apolipoprotein L1	0.01984183
ARSA	Arylsulfatase A	− 0.0645501
ATG10	Autophagy Related 10	0.29116592
ATG12	Autophagy Related 12	0.41732508
ATG4A	Autophagy Related 4A Cysteine Peptidase	− 0.4109275
BCL2L1	BCL2 Like 1	0.08080018
BNIP3L	BCL2 Interacting Protein 3 Like	− 0.0593609
CAPNS1	Calpain Small Subunit 1	0.29692815
CX3CL1	C-X3-C Motif Chemokine Ligand 1	− 0.0257955
DAPK2	Death Associated Protein Kinase 2	− 0.0054654
EEF2K	Eukaryotic Elongation Factor 2 Kinase	− 0.1395836
EIF2S1	Eukaryotic Translation Initiation Factor 2 Subunit Alpha	0.11957601
EIF4G1	Eukaryotic Translation Initiation Factor 4 Gamma 1	0.25277625
GNAI3	G Protein Subunit Alpha I3	0.05925382
KLHL24	Kelch Like Family Member 24	− 0.2001073
MAPK8IP1	Mitogen-Activated Protein Kinase 8 Interacting Protein 1	− 0.016518
MBTPS2	Membrane Bound Transcription Factor Peptidase, Site 2	0.19392453
NLRC4	NLR Family CARD Domain Containing 4	− 0.0950729
PRKCD	Protein Kinase C Delta	− 0.2369349
RELA	RELA Proto-Oncogene, NF-KB Subunit	0.18438262
SIRT2	Sirtuin 2	− 0.0924301
SPHK1	Sphingosine Kinase 1	0.10961033
ST13	ST13 Hsp70 Interacting Protein	0.29078597

**Fig. 2:**
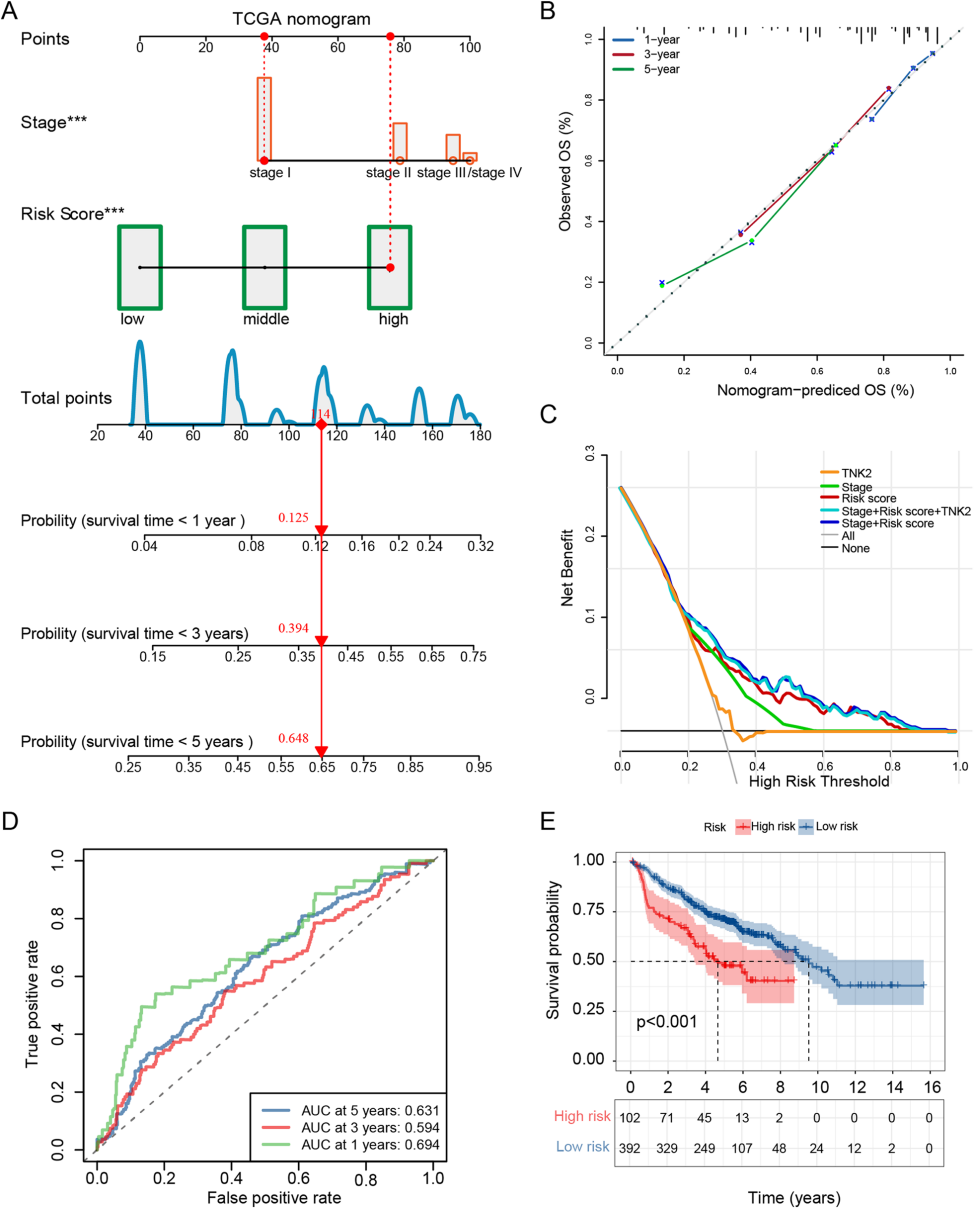
Clinical significance of the risk signature in the TCGA-LUAD cohort and validation in GEO cohorts. **A** The construction of a nomogram by integrating risk score and clinical stage. **B** The calibration plots compare the observed (y-axis) and predicated (x-axis) 1-, 3- and 5-year survival. **C** Decision curve analysis revealed that the combination of the risk score and TNM stage improved net benefit at any given threshold. The predicting accuracy of the 23-gene signature was tested in 495 patients with LUAD collected from GSE31210, GSE37745, and GSE50081 cohorts, by plotting ROC (**D**) and Kaplan–Meier survival curves (**E**)

We also constructed a nomogram prediction model with stage and risk score (concordance index = 0.714) (Fig. 2A). As shown in calibration curves, the observed and predicted probabilities of patient groups are located along the 45-degree line, suggesting high predictive accuracy of the nomogram (Fig. 2B). Decision Curve Analysis (DCA) is a novel method to assess the clinical net benefit of predictive models, treatments, diagnostic tests, and molecular markers. We found that the combination of the stage with a risk score was the preferred model because it showed the best net benefit at all given thresholds (Fig. 2C). We further validate the prognostic performance of the 23-gene signature with 495 patients with LUAD collected from three GEO datasets (GSE31210, GSE37745, and GSE50081). In this validating set, the risk score could distinguish patients with better outcomes (Fig. 2D, E). A nomogram was also constructed with a C-index of 0. 611, followed by a calibration curve (Additional file 2: Fig. S2A, B).

**Fig. 3:**
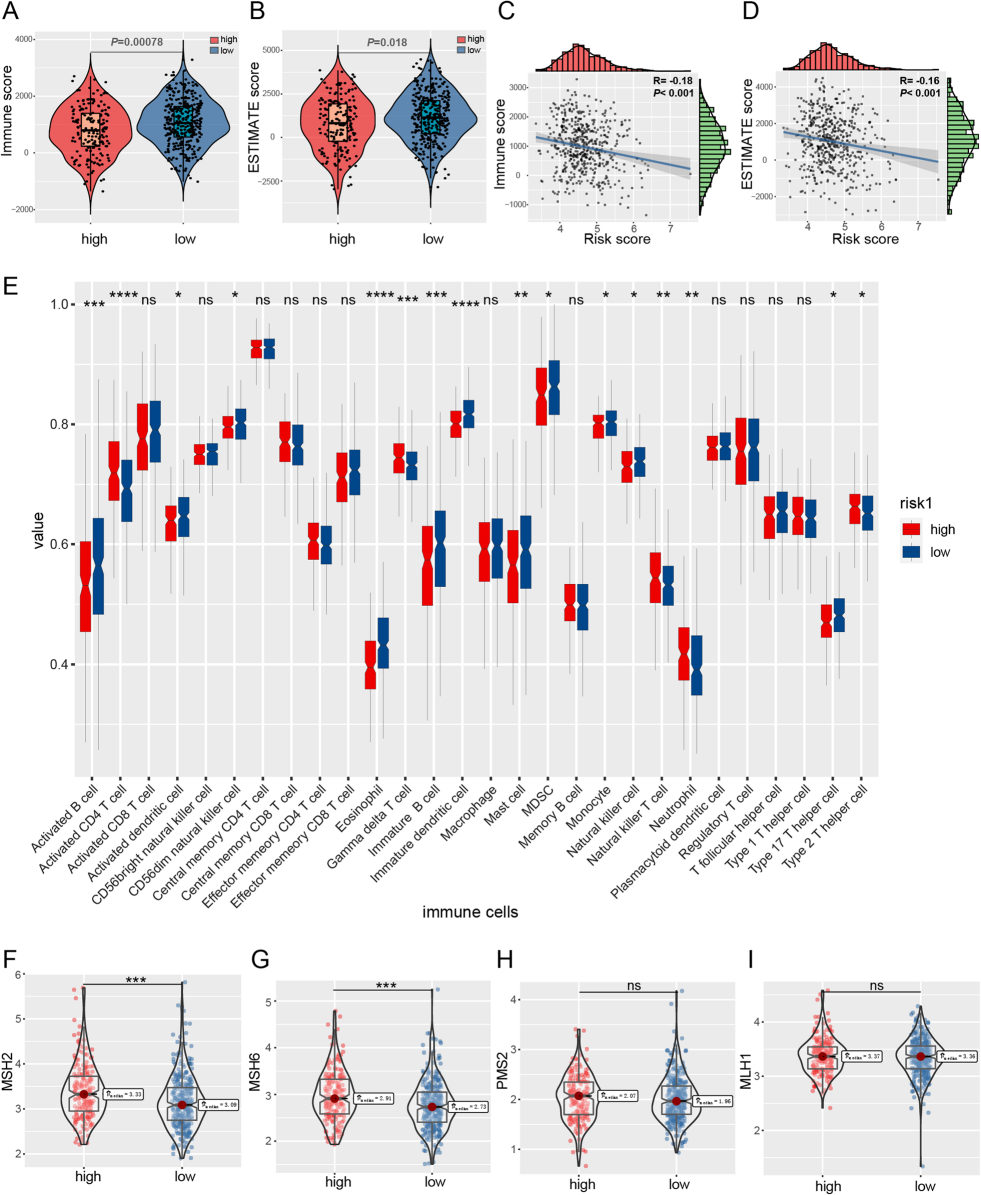
Correlation between risk score and tumor immunogenicity in the TCGA-LUAD cohort. ESTIMATE algorithm indicates that immune (**A**) and ESTIMATE (**B**) scores in the high-risk group are significantly lower than those in the low-risk group. Immune (**C**) and ESTIMATE (**D**) scores are negatively associated with the risk score. **E** Comparison of the composition of 28 immune cell subsets in LUAD between the high- and low-risk groups. **F**–**I** In the low-risk group, there were decreased expression levels of mismatch repair genes, MSH2 and MSH6

Moreover, we found that both the immune score and ESTIMATE score were significantly lower in the high-risk group than in the low-risk group (Fig. 3A, B), which was confirmed by the negative association of immune score and ESTIMATE score with a risk score (Fig. 3C, D). While using ssGSEA to identify 28 types of immune cells in LUAD based on previously reported signature genes, we found that the predicting signature could discriminate the differences in immune cell infiltration between the high- and low-risk group (Fig. 3E). A deficient DNA mismatch repair (MMR) system was positively associated with sensitivity to immune checkpoint blockade (Luchini et al. 2019). MSH2, MSH6, PMS2, and MLH1 are often examined to evaluate the mismatch repair deficiencies (Le et al. 2017). Our results indicated that the low-risk group had significantly lower expression levels of mismatch repair genes, MSH2 and MSH6 (Fig. 3F–I), indicating a compromised DNA mismatch repair ability. Increased expression levels of CTLA4 and defective MMR suggest the low-risk group is more likely to benefit from immunotherapy.

**Fig. 4:**
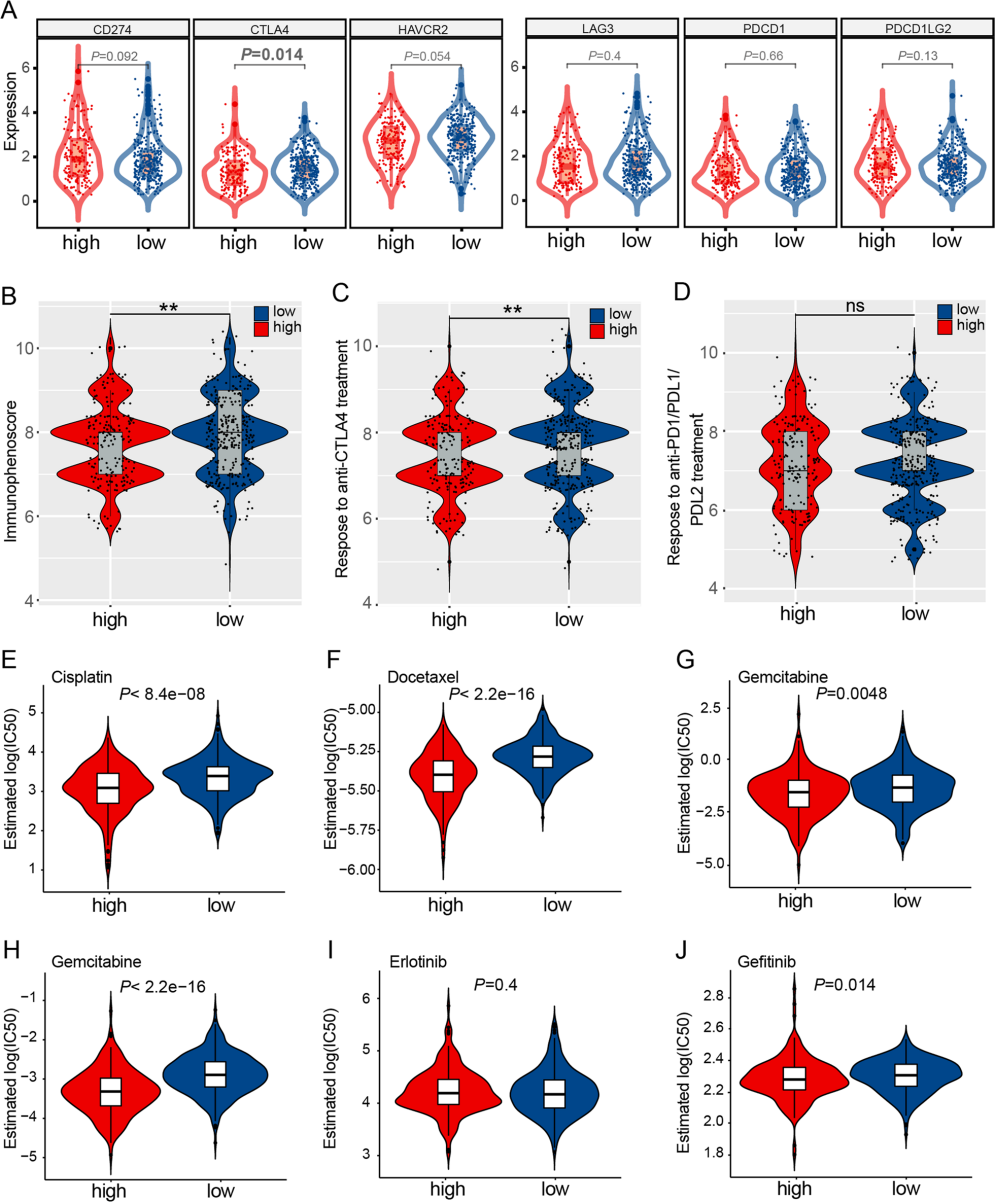
The risk signature predicted response to common antitumor drugs in the TCGA-LUAD cohort. **A** Comparison of immune checkpoint gene expression between the high- and low-risk groups. **B**–**D** Calculation of the immunophenoscore **B** and the sensitivity to anti-CTLA4 **C** and anti-PD1/PDL1/PDL2 **D** immunotherapy for the two subgroups. **E**–**J**. Half inhibitory concentrations (IC50) were compared between high- and low-risk groups for cisplatin (**E**), docetaxel (**F**), gemcitabine (**G**), paclitaxel (**H**), erlotinib (**I**), and gefitinib (**J**)

We also interrogated the differences in immune checkpoint gene expression between high- and low-risk groups, including programmed cell death 1 ligand 1 (PD-L1)/CD274, cytotoxic T-lymphocyte associated protein 4 (CTLA4), hepatitis A virus cellular receptor 2 (HAVCR2), Lymphocyte-Activation Gene 3 (LAG3), Programmed Cell Death 1 (PDCD1), and programmed cell death 1 ligand 2 (PDCD1LG2). Interestingly, CTLA4 expression levels were significantly high in the low-risk group than in the high-risk group (Fig. 4A). Immunophenoscore (IPS) is the most comprehensive immune determinant by far. The low-risk group showed significantly enhanced IPS when compared with the low-risk group. Moreover, The low-risk group was more likely to respond to anti-CTLA4 antibodies than the high-risk group (Fig. 4B–D). Finally, we investigated whether the risk score is related sensitivity of commonly used anticancer drugs. Estimated IC50 indicated that the high-risk group has significantly lower IC50 for cisplatin, docetaxel, gefitinib, gemcitabine, and paclitaxel (Fig. 4E–J). These results suggested that LUAD patients in the high-risk group were more like to respond to these drug treatments.

### Inhibiting ACK1 reduced proliferation and promoted apoptosis of NSCLC cells

**Fig. 5:**
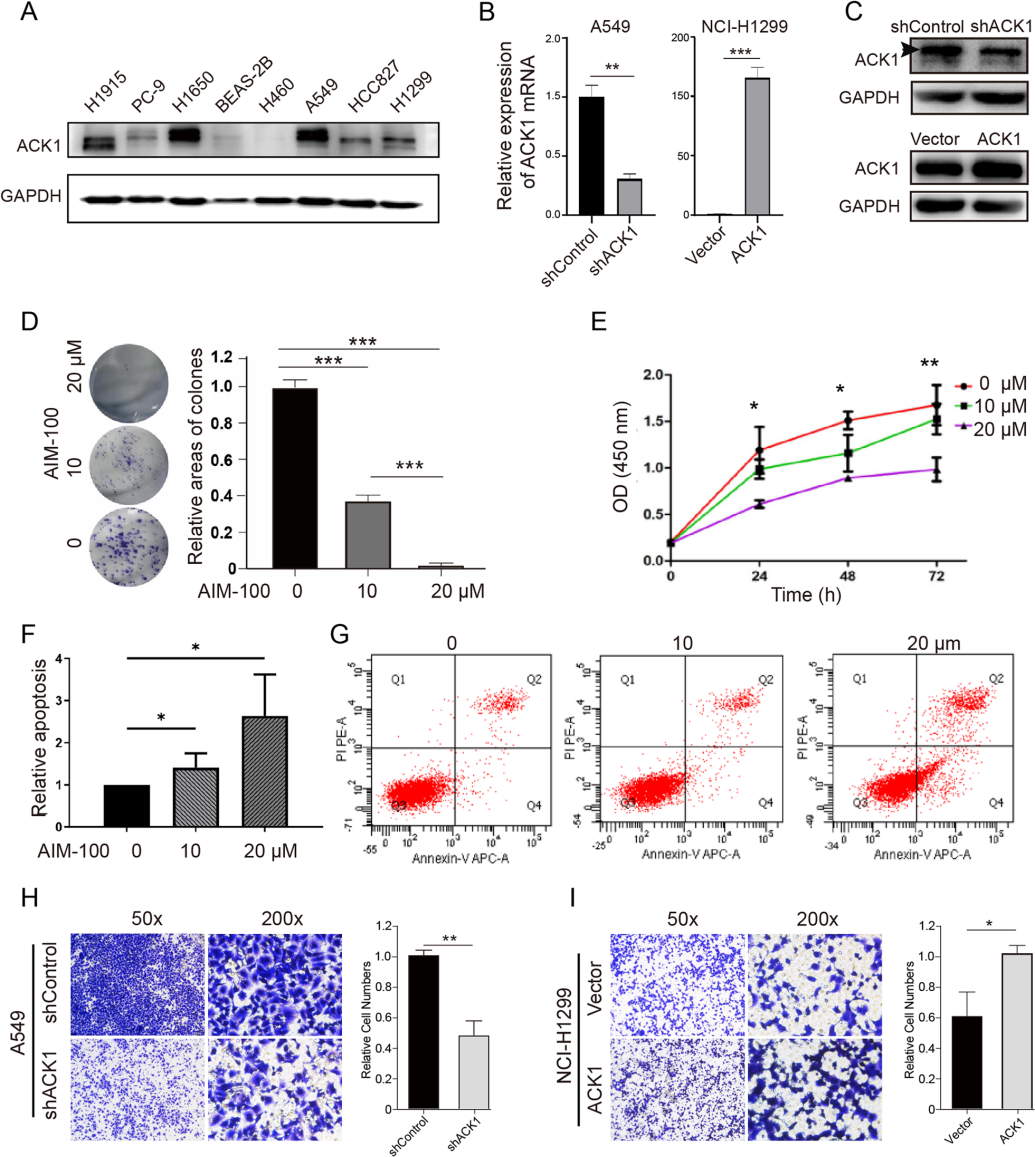
The function of ACK1 in lung cancer cells. **A** Expression of ACK1 in human lung bronchial epithelial (Beas-2B) and indicated lung cancer cell lines. **B**, **C** RT-qPCR **B** and western blot analyses **C** were used to verify ACK1 at mRNA and protein levels in A549 cells infected with lenti-shACK1 and NCI-H1299 cells with empty vectors and ACK1 overexpression lentiviral vectors. The black arrowhead indicated the ACK1 bands in the blot. The colony formation **D** and CCK8 **E** assays showed the effect of ACK1 inhibitor (AIM-100) on A549 cell viability and proliferation capacity, respectively. Cells were allowed to grow for ten days to form visible colonies. **F**, **G** The impact of AIM-100 on apoptosis of A549 cells at 24 h after treatments. **H**, **I** After an incubation of 18 h, the effects of knockdown and overexpression of ACK1 on cell migration were measured using a transwell assay in A549 **H** and NCI-1299 **I** NSCLC cells, respectively. *P < 0.05, **P < 0.01, ***P < 0.001

We next validated the effects of ACK1 using in vitro experiments. We first determined the expression levels of ACK1 in different NSCLC cell lines (Fig. 5A). Based on endogenous ACK1 expression levels, A549 and NCI-H1299 cells were used to knock down and overexpress the ACK1 gene, respectively, followed by RT-qPCR (Fig. 5B) and western blot (Fig. 5C) assays to confirm the genetic manipulation efficiency. AIM-100, an ACK1 inhibitor, significantly reduced the colony formation of A549 cells (Fig. 5D). Inhibition of ACK1 also significantly decreased A549 cell proliferation (Fig. 5E). In contrast, AIM-100 significantly promoted A549 cell apoptosis as quantitated by flow cytometry (Fig. 5F, G). Moreover, we observed that silencing ACK1 suppressed the migration of A549 cells (Fig. 5H); meanwhile, overexpression of ACK1 accelerated the movement of NCI-H1299 cells (Fig. 5I).

### Inhibiting ACK1 induced autophagy-like response

**Fig. 6:**
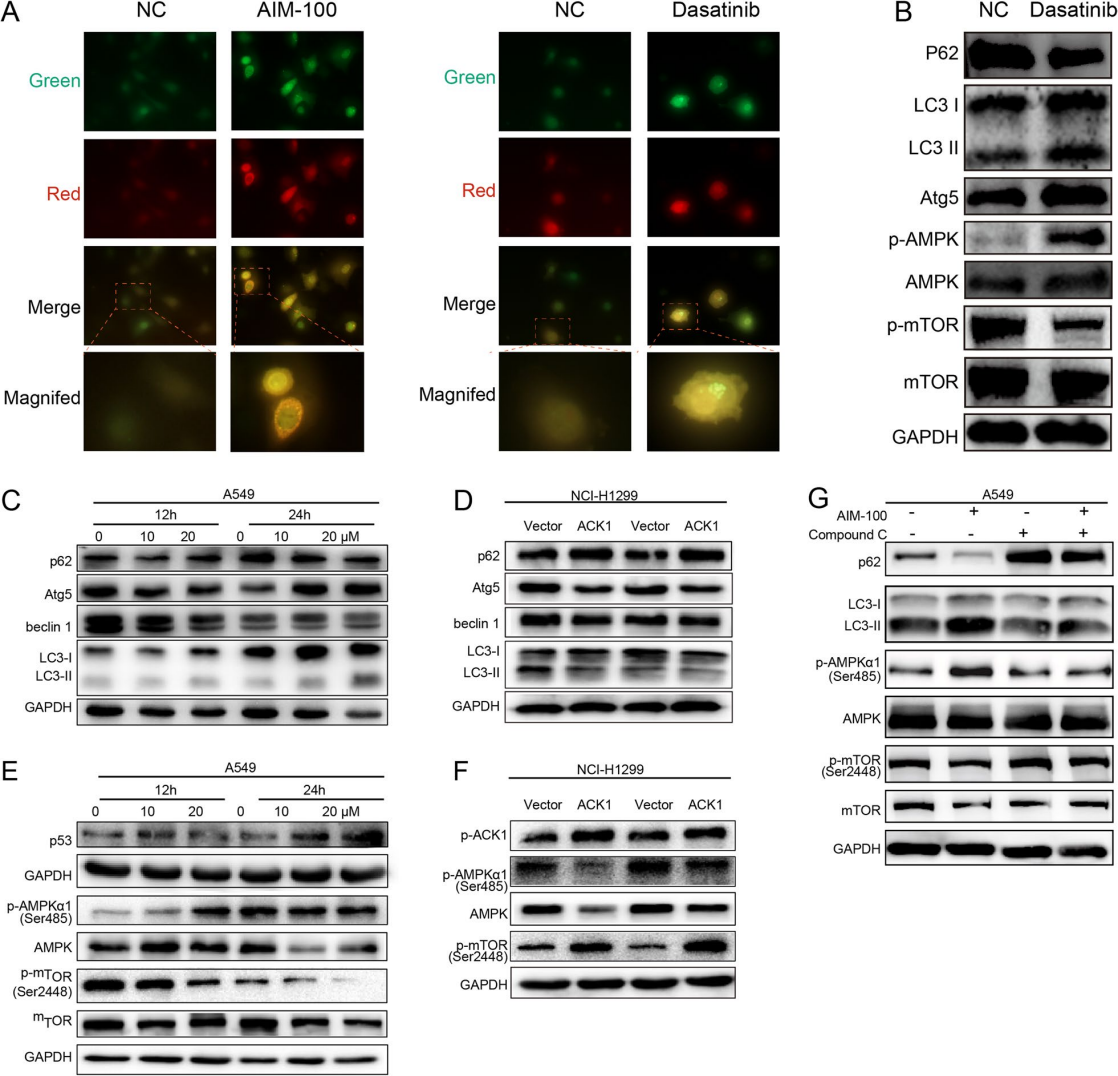
Suppressing ACK1 stimulated an autophagy-like response and activated the AMPK/mTOR pathway in lung cancer cells. **A** A549 cells were infected with adenoviral vectors carrying mRFP-GFP-LC3 and cultured for 24 h, followed by incubation with AIM-100 (20 μM) or Dasatinib (20 μM) for an additional 12 h. mRFP-LC3 and GFP-LC3 puncta, representing autophagosomes, in A549 cells induced by AIM-100 or Dasatinib (at × 400 magnification). The number of LC3 puncta per cell was quantitated. **B** A549 cells were treated with Dasatinib (20 μM) for 12 h. Cell lysates were analyzed, and immunoblots were probed with indicated antibodies. **C**, **E**) A549 cells were treated with different concentrations of AIM-100 for indicated hours. Influences of AIM-100 on common biomarkers of autophagy **C** and the AMPK/mTOR signaling pathway **E** were examined. **D**, **F** NCI-H1299 cells were used to investigate the effects of ACK1 overexpression on the biomarkers of autophagy (**D**) and the AMPK/mTOR signaling (**F**). **G** A549 cells were incubated with AIM-100 (20 μM) and/or AMPK inhibitor (Compound C, 20 μM) for 12 h, succeeded by Western blot. *P < 0.05, **P < 0.01, ***P < 0.001

Furthermore, we tested whether inhibiting ACK1 induces autophagy in NSCLC cells. Besides AIM-100, Dasatinib, another ACK1 inhibitor, has been frequently used to inhibit ACK1 both in vitro and in vivo (Zhang et al. 2020). Here, we adopted Dasatinib to validate the effects of AIM-100 on NSCLC cells. First, A549 cells were infected with retroviral vectors overexpressing the mRFP-GFP-LC3 fusion protein. Using a fluorescence microscope, we observed yellow and red LC3 puncta in A549 cells treated with either AIM-100 or Dasatinib (Fig. 6A). Second, western blot analysis was performed to confirm the effects of ACK1 inhibition on autophagy-like response. It is known that once autophagy is initiated, microtubule-associated protein 1 light chain 3 β (MAP1LC3B) is cleaved, named LC3-I, which is conjugated with lipid phosphatidylethanolamine (PE) to form LC3-II. The resulting lipid-conjugated forms of LC3 are further recruited to autophagosome membrane. As a result, LC3-II levels increase with the number of autophagosomes and are therefore used to measure autophagy activity quantitatively despite some limitations (Klionsky et al. 2021; Levy et al. 2017). We found that Dasatinib increased autophagy-related protein 5 (Atg5) and processed LC3-II form coupled with a decrease in p62 (Fig. 6B). P62 is a cargo protein that disintegrates during the progression of autophagy flux. Likely, 24 h after AIM-100 treatment, increased LC3-II, Atg5, and decreased p62 levels were observed in A549 cells. (Fig. 6C and Additional file 4: Fig. S4). The decrease of cargo protein p62 was observed as early as 12 h after ACK1 inhibition. The time course of autophagy-like events induction by ACK1 inhibitor was also investigated. Both low and high concentrations of AIM-100 could induce autophagy-like reponses 12 h after treatment. We also checked essential proteins that regulate the autophagic process. Intriguingly, suppression of ACK1 increased Atg5 but not beclin 1 (Fig. 6C). Inversely, we found that ACK1 overexpression suppressed autophagy-like reponses, as evidenced by decreased LC3-II and increased p62 in NCI-1299 cells (Fig. 6D and Additional file 4: Fig. S4). These results suggest that inhibition of ACK1 enhanced autophagy-like reponses in A549 cells.

### ACK1 inhibitor activated the AMPK signaling pathway

Our RNA-seq results revealed that silencing of ACK1 affected many fundamental signaling pathways, among which AMPK is known to regulate autophagy (Additional file 3: Fig. S3). As exhibited in Fig. 6B, Dasatinib augmented the phosphorylation of AMPK (Ser485) but depressed the phosphorylation of mTOR (Ser2448) 12 h post-treatment. Likely, AIM-100 also activated the AMPK-mTOR signaling pathway at 12 h; however, its effects on AMPK phosphorylation were not captured after exposure to AIM-100 for 24 h (Fig. 6E and Additional file 4: Fig. S4). Furthermore, enforced expression of ACK1 inactivated the AMPK/mTOR signaling pathway in NCI-H1299 cells (Fig. 6F and Additional file 4: Fig. S4). Moreover, Compound C (CC) abolished AIM-100-induced AMPK phosphorylation and mTOR dephosphorylation, restoring levels of p62 and LC3-II altered by AIM-100 (Fig. 6G and Additional file 4: Fig. S4). These results suggested that the ACK1 inhibitor activated the AMPK signaling pathway.

### Inhibiting AMPK sensitized lung cancer cells to ACK1 inhibitor

**Fig. 7:**
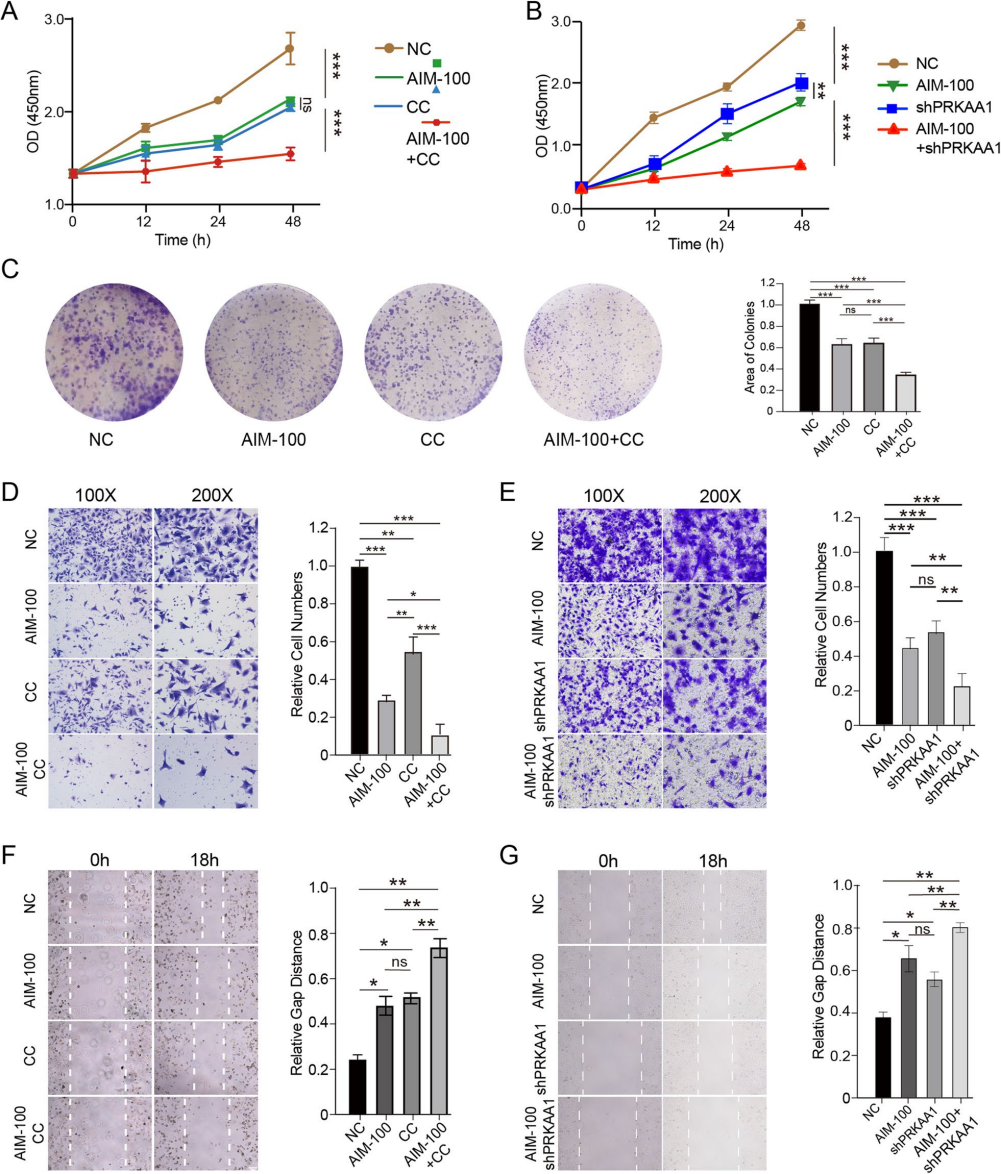
AMPK inhibitor compound C (CC, 20 μM) and shPRKAA1 reinforced the cytotoxic effects of AIM-100 (20 μM) on A549 cells. **A** A CCK8 assay for A549 cells treated with AIM-100, CC, or both agents for 48 h. **B** Impacts of AIM-100, shPRKAA1, or combined treatments on the proliferation of A549 cells). **C** The colony formation assay was adopted to estimate the influences of indicated treatments on A549 cell proliferation. Colony formation was estimated after cells were treated for ten days. **D**–**G** Wound-healing (**D**, **E**) and transwell migration assays (**F**, **G**) were used to check the effects of indicated treatments on A549 cells’ migration abilities, which were evaluated after cells were treated with indicated agents for 18 h. *P < 0.05, **P < 0.01, ***P < 0.001

We also would like to know whether the AMPK activation induced by inhibiting ACK1 was protective or detrimental for NSCLC cells. Our results showed that inhibiting AMPK by CC significantly increases lung cancer cells’ response to AIM-100, as revealed by CCK8, colony formation, wound healing, and transwell assays (Fig. 7A, C, D, F). Besides, we also used shRNA to knock down *protein kinase AMP-activated catalytic subunit alpha 1* (*PRKAA1*), which encodes a catalytic subunit of AMP-activated protein kinase (AMPK). Like CC, silencing *PRKAA1* sensitized lung cancer cells to AIM-100 as shown by CCK8, transwell, and wound healing analyses (Fig. 7B, E, G).

**Fig. 8:**
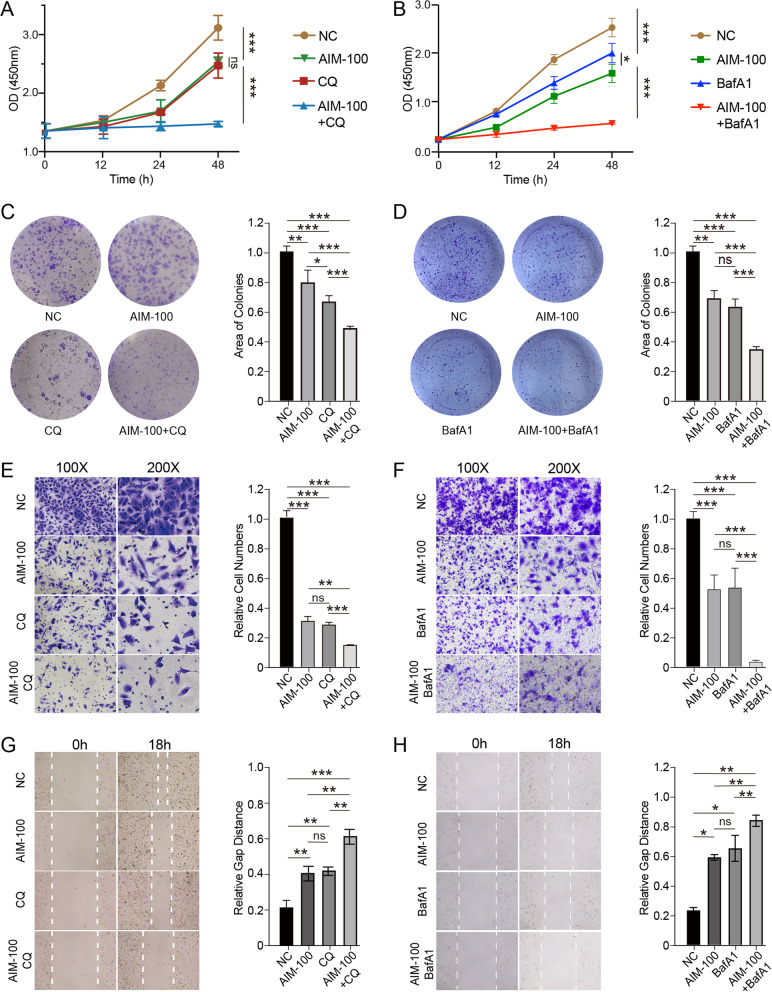
Chloroquine (CQ, 20 μM) or Bafilamycine A1 (BafA1, 20 nM) sensitized lung cancer cells to ACK1 inhibition by AIM-100 (20 μM). **A** A549 cells were treated with CQ, AIM-100, or in combination for 48 h, and cellular viability was measured using CCK8 assay. **B** CCK8 assay was performed on A549 cells with the replacement of CQ with BafA1. **C**, **D** After A549 cells were treated as **A** and **B** for ten days, colony formation was evaluated for each group. **E**–**H** Impacts of indicated treatments on A549 cells’ migration abilities were assessed by wound-healing (**E**, **F**) and transwell migration assay (**G**, **H**), which were determined after exposure to noted drugs for 18 h. *P < 0.05, **P < 0.01, ***P < 0.001

Given that inhibiting ACK1 provoked an autophagy-like response, we speculated that the resulting response might attenuate the efficacy of therapies targeting ACK1. If so, blocking autophagy-like response should sensitize lung cancer cells to ACK1 inhibitors. Cells were treated with both AIM-100 and a lysosomal degradation inhibitor (chloroquine, CQ) or two agents alone. CCK8 results revealed that ACK1 inhibitor combined with CQ significantly more potently suppressed proliferation, migration, and wound healing of lung cancer cells than either AIM-100 or CQ alone. Our results indicate that AIM-100 induced protective autophagy-like response, thereby causing A549 cells to be insensitive to the therapy, while the combination therapy can overcome resistance to the ACK1 inhibitor. CQ, in combination with the ACK1 inhibitor, significantly inhibited viability, proliferation (Fig. 8A, C), and migration (Fig. 8E, G) of A549 cells compared with either agent alone. Bafilomaycin A1 (BafA1), an inhibitor of lysosomal degradation, was also adopted to block autophagy-like response and validate the beneficial effects of dual inhibitors (Fig. 8B, D, F, H). Similarly, our results indicated that combing BafA1 and AIM-100 more potently reduced proliferation and migrations of A459 cells than either inhibiting autophagy-like response or ACK1. Overall, Blocking autophagy-like response with inhibitors of lysosomal degradation aggravated the inhibitory effects of AIM-100 on lung cancer cells.

### Dasatinib and CQ combination treatment reduces tumor burden in an NSCLC xenograft model

**Fig. 9:**
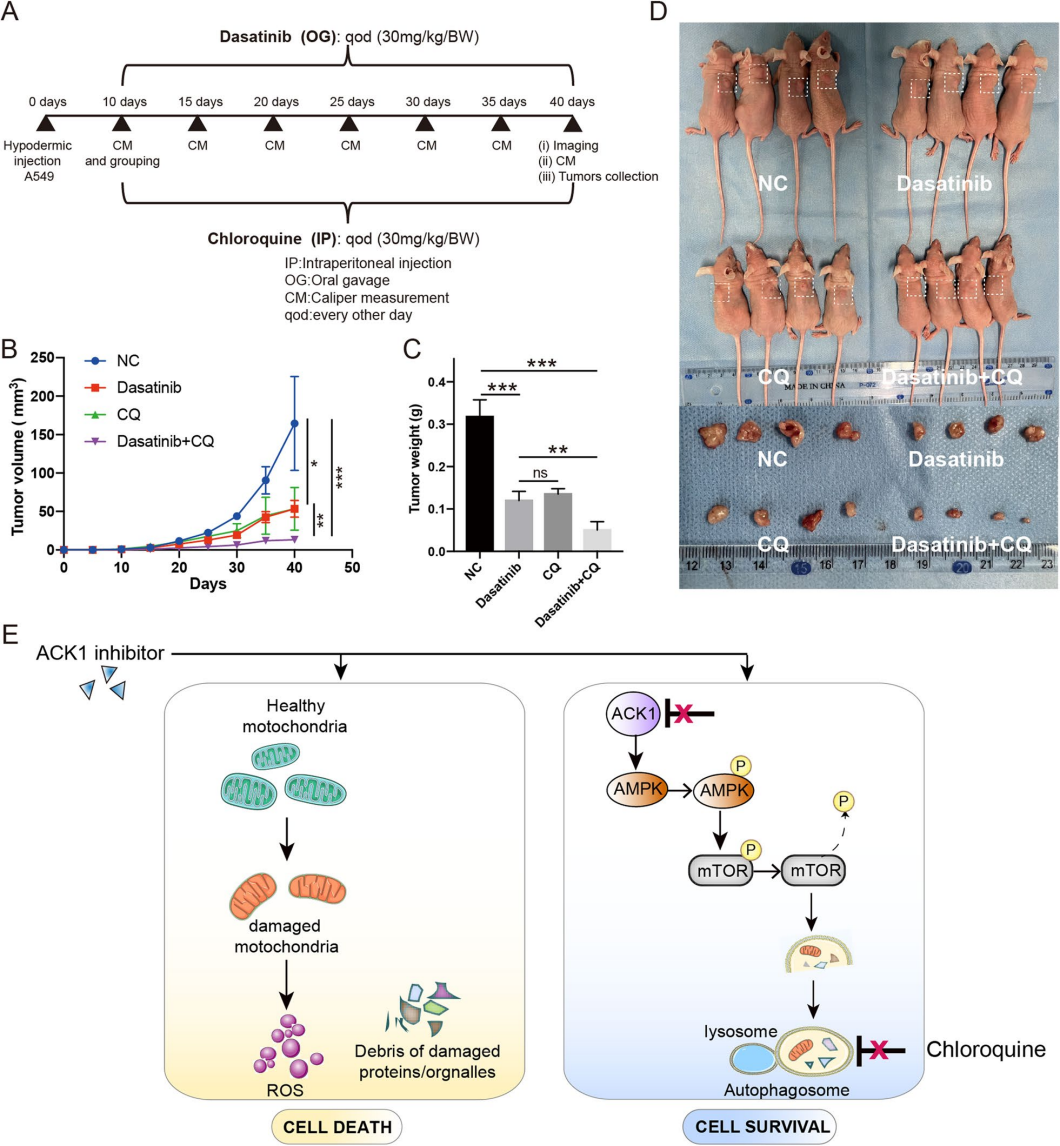
The combination of Dasatinib and chloroquine reduced tumor volume and weights in an A549 cell-derived xenograft model. **A** A Schematic diagram of the animal study. **B** Caliper measurement of tumor volumes for different groups at the indicated time points. **C** The average weight of mice was measured at the endpoint. **D** The upper row exhibits mice administrated with vehicle control; Dasatinib, chloroquine (CQ), and a combination of Dasatinib and CQ. The lower row shows tumors collected from corresponding mice. **E** The schematic mechanism on the left panel showed that the ACK1 inhibitor killed tumor cells by partially impairing cellular organelles and proteins. Damaged organelles lead cells to death by partly generating reactive oxygen species (ROS). The left panel indicated that upon treating the ACK1 inhibitor, the adaptive autophagy-like response cleared damaged cellular organelles and proteins, eradicated oxidative damages, and provided nutritional needs, consequently maintaining homeostasis and cell survival. CQ inhibited ACK1 inhibitor-triggered protective autophagy-like response. *P < 0.05, **P < 0.01, ***P < 0.001

Based on in vitro results, we verified the antitumor efficacy of the combined ACK1 inhibitor and CQ in vivo using a subcutaneous NSCLC xenograft model. Dasatinib and CQ were used to inhibit ACK1 and autophagy-like response, respectively, because they were proven by FDA. A study schema is displayed in Fig. 8A. Dasatinib alone led to a significant decrease in the growth and weight of NSCLC xenograft, to a certain extent. Moreover, mice administrated with both CQ and Dasatinib exhibited significantly reduced tumor burden as measured by tumor volumes and weights compared to counterparts receiving single drugs (Fig. 9A–D). These results further demonstrated that inhibiting autophagy-like response by CQ enhances NSCLC’s response to the ACK1 inhibitor. Overall, the ACK1 inhibitor activated protective autophagy-like response, which decreased the antitumor efficacy of the ACK1 inhibitor. However, the blockade of autophagy-like response improved the therapeutic effects of the ACK1 inhibitor (Fig. 9E).

## Discussion

NSCLC is one of the most lethal malignancies worldwide. Targeted therapies have dramatically improved life quality and survival in patients harboring druggable oncogenic driver mutations. However, responses to these therapies are mostly partial and short-term. For instance, sometimes, patients may experience a moderate response to anticancer agents; however, some tumor cells can undergo early adaptive changes that make themself resistant to therapy and survive. The activities involved in adaptive resistance include, but are not limited to, the activation of survival and anti-apoptotic signals, histological transformation (e.g., the transformation of NSCLC to a small-cell lung cancer histology), phenotypic change (e.g., epithelial-mesenchymal transition (EMT)), and autophagy (Holohan et al. 2013). Discerning resistance-related events and developing a combination regime is a promising strategy to advance outcomes in patients with NSCLC.

ACK1 is a promising target in cancer treatment. However, inhibiting ACK1 has archived only a moderate response in cancer patients. The underpinning mechanism of ACK1 inhibitor resistance remains obscure. The current study showed that inhibiting ACK1 alone hindered proliferation and migration but promoted apoptosis of lung cancer cells. The oncogenic roles of ACK1 have been verified in various cancers. ACK1 can promote cancer cell proliferation, EMT, migration, and invasion in different cancers, including colorectal cancer, hepatocellular carcinoma, glioma, prostate cancer, renal cancer, and NSCLC (Chua et al. 2010; Hu et al. 2016; Lei et al. 2015; Mahajan et al. 2013, 2005; Qi and Ding 2018; Tan et al. 2014; Zhang et al. 2015). Many studies substantiated that ACK1 inhibitors can inhibit tumor cells. For example, Tan et al. demonstrated that Bosutinib downregulated migration and invasion of Kras mutant NSCLC cells via neutralizing ACK1 (Tan et al. 2014). Recently, Zhang et al. identified ACK1 activation as a novel mechanism resisting EGFR inhibitor ASK120067 in NSCLC and concurrently inhibiting EGFR and ACK1 could overcome the acquired resistance of ASK120067 efficaciously (Zhang et al. 2020).

The clinical relevance of ACK1 has been investigated in NSCLC. ACK1 expression levels were significantly higher in 210 Singaporean lung adenocarcinomas than in paired adjacent non-tumor tissues. Intriguingly, ACK1 expression in adjacent tissue was significantly associated with prognosis but not in the tumor itself (Tan et al. 2014). Hu et al. verified significantly elevated expression of ACK1 in NSCLC tissue at both transcript and protein levels compared with paired normal tissues (Chua et al. 2010). They also reported a reversal associated between ACK1 expression levels and survival time (Chua et al. 2010). The controversies over the prognostic value of ACK1 suggest that ACK1 alone may not be sufficient to predict the clinical outcomes in NSCLC. Alternatively, accumulating evidence substantiated that the combination of molecular biomarkers with their cancer-related functional relevance may greatly improve compared to a single molecule (Sheng et al. 2020; Zhu et al. 2021a, b).

Since our RNA-seq results suggested that ACK1 was related to autophagy, we built a signature of 23 ACK1-associated autophagy genes by integrating ACK1 and its functional role in autophagy. The risk score derived from this signature was superior to ACK1 alone in predicting prognosis in LUAD. We also construct a nomogram with refined discrimination ability. DCA illustrated that the risk score combined with TNM stage and ACK1 attained the highest net benefit among all tested parameters. We integrated ACK1 and autophagy because of two reasons. First, autophagy is tightly linked to NSCLC, and characteristic patterns of autophagy-related genes in NSCLC are significantly associated with clinical outcomes of patients (Liu et al. 2019). Second, our findings revealed that autophagy resulted in adaptive resistance of the ACK1 inhibitor in lung cancer cells, suggesting the critical role of ACK1 in regulating autophagy. Our results agree with several other publications (Sheng et al. 2020; Zhu et al. 2021a, b). Shen et al. proclaim that a signature consisting of cancer survival genes could robustly predict lung cancer progression (Sheng et al. 2020). In addition, an oncogene UBE2T promoted cisplatin resistance by inducing protective autophagy in lung cancer cells, and the signature of UBE2T-related autophagy genes showed better prognostic accuracy than UBE2T alone or traditional TNM stage (Zhu et al. 2021a, b).

Interestingly, our ACK1-associated gene signature could also predict response to ICIs, chemotherapy, and targeted therapy in LUAD. The low-risk group showed increased expression levels of CTLA4, suggesting a response to ICIs. Moreover, significantly decreased expression levels of MSH2 and MSH6 were observed in the low-risk group when compared with the low-risk group. Generally, defective mismatch repair in cancer is speculated to lead to a dramatic increase in mutation-associated neoantigens (MANAs) recognizable by the immune system. Several studies demonstrated that MMR deficiency-related mutations are predictive of the response to PD-1 blockade in colorectal cancer (Le et al. 2017; Overman et al. 2018). Furthermore, Le and colleagues launched a clinical trial across 12 different tumor types, substantiating that patients with advanced mismatch repair-deficient cancers greatly benefited from PD-1 blockade therapy regardless of cancer type (Le et al. 2017). In contrast, patients in the high-risk group tended to respond to several chemotherapeutic agents. These results are in line with other studies. Patients in the low-risk group may be more suitable for ICIs, while those in the high-risk group may be more likely to respond to chemotherapy.

Furthermore, our in vitro and in vivo studies showed that the ACK1 blockade spurred autophagy-like response, and blocking autophagy-like response enhanced the antitumor efficacy of ACK1 inhibitors. Autophagy-like event seemed to be an adaptive response to the ACK1 tyrosine kinase inhibitor. While exposed to nonfatal stress, such as alterations in temperature, hypoxia, redox potential, extracellular signals, and cytotoxic agents, cells experience metabolic and physiological reshaping to restore disrupted homeostasis and resist death. The adaptive response mechanisms include, but be not limited to, autophagy, endoplasmic reticulum (ER) stress signaling, and senescence (Chern and Tai 2020). The role of autophagy in the tumor is context-dependent. Some reported that autophagy provoked by antineoplastic therapies aggravated tumor cell death, while others showed that induced autophagy provided tumor cells with survival advantages to fight stresses and acted as a self-protection reaction for resisting therapy. In the latter case, autophagy facilitates the survival of tumor cells under cytotoxic stress by scavenging impaired organelles, minimizing oxidative damages, and fulfilling nutritional needs (Chern and Tai 2020). Many antitumor drugs unintentionally induced adaptive autophagy in cancer cells, including targeted therapeutic agents (gefitinib, dasatinib, lapatinib, trametinib, and trastuzumab) and chemotherapeutic agents (e.g., 5-Fluorouracil, cisplatin, docetaxel, and vincristine) (Chern and Tai 2020). In our study, autophagy is part of an adaptive response conferring acquired resistance to ACK1 inhibitors in lung cancer cells. Consistent with our findings, mounting studies manifested that autophagy inhibition boosted the cytotoxic effects of targeted therapies in NSCLC (Chude and Amaravadi 2017; Kwon et al. 2019; Tang et al. 2018). Many targeted drugs can synergize with anti-autophagic drugs to augment antitumor efficiency in NSCLC, including lapatinib, afatinib, erlotinib, gefitinib, and dacomitinib (Kwon et al. 2019). Cepharanthine, a novel autophagy inhibitor, sensitizes NSCLC cells to EGFR-TKI dacomitinib by preventing autophagosome-lysosome fusion (Tang et al. 2018). Similarly, chloroquine intensified erlotinib-induced growth inhibition in lung cancer cells (Zou et al. 2013). To date, many clinical trials are ongoing, investigating whether hydroxychloroquine, the clinically approved autophagy inhibitor, leads to accelerated tumor regression in combination with available drugs across a broad spectrum of cancer (Mahajan et al. 2010). Taken together, these findings indicate that inhibiting autophagy-like response increases the cytotoxicity of ACK1 inhibitors in cancer cells by overcoming drug-induced adaptive resistance.

We found that the ACK1 inhibitor promoted protective autophagy-like response via the AMPK/mTOR signaling pathway. In line with our findings, Li et al. found that high mobility group box protein 1 (HMGB1) mediated doxorubicin (DOX) resistance in human hepatocellular carcinoma cells (HCCs). The underlying mechanism was that the HMGB1 activated the AMPK/mTOR signaling pathway to invoke adaptive autophagy (Li et al. 2021). Inhibition of either HMGB1 or autophagy conquers the resistance of HCCs to DOX (Li et al. 2021). mTOR is a central checkpoint of autophagy. Activation of mTOR negatively regulates autophagy, whereas anticancer drugs suppressing the PI3K/Akt/mTOR pathway can trigger autophagy (Galluzzi et al. 2017; Janku et al. 2011). The AMPK pathway is one of the crucial pathways controlling autophagic activity under stress (Yang et al. 2009). Some studies suggested that the AMPK/mTOR signaling pathway was implicated in p53-mediated autophagy (Tasdemir et al. 2008). Tasdemir et al. found that cytoplasmic p53 downregulated autophagy by suppressing the AMPK/mTOR pathway (Tasdemir et al. 2008). Likewise, a recent publication revealed that the knockdown of UBE2T resulted in cytoplasmic translocations of p53, which in turn decreased autophagic activity through the AMPK/mTOR pathway (Zhu et al. 2021a, b). Activation of AMP-activated protein kinase (AMPK) can cause the inactivation of mTORC1 and consequently inhibit the autophagic process. Moreover, AMPK also enhances autophagic activity by phosphorylating and activating ULK1 and beclin 1 (BECN1) (Galluzzi et al. 2017). However, it is unclear how the ACK1 inhibitor activates the AMPK signal pathway. Since nutrient energy sensor AMP kinase (AMPK) is sensitive to cAMP accumulation resulting from ATP consumption, it may be activated by metabolic stresses caused by anticancer therapy (Holohan et al. 2013).

The limitations of the current study should be addressed. First, the predictive capacity of our multi-gene model should be tested in patients with lung adenocarcinoma in the future. Second, in addition to chemical approaches, such as autophagy inhibitors, genetically targeting the autophagic machinery should be conducted to strengthen the findings. Third, the autophagy-like response should be validated and quantified using different methods, such as mRFP-GFP-LC3B fusion protein assay coupled with ratiometric FACS analysis. Finally, most of the experiments were conducted in A549 cells only. Extra NSCLC cell lines should be used to verify these results. The findings should be interpreted cautiously.

## Conclusions

We found that the ACK1 inhibitor triggered an adaptive autophagy-like response in lung cancer cells, and blocking AMPK or lysosomal degradation increased the cytotoxic effects of ACK1-targeted therapy. Moreover, integrating ACK1 and its regulation on autophagy produced a robust predicting signature for survival and drug sensitivity in LUAD. Overall, these results provide evidence of a potential role for autophagy in therapy evasion.

## Supplementary Information

**Additional file 1: Fig. S1.** Establishment of a prognostic signature of ACK1-correlated autophagy genes using the LASSO regression model in the TCGA-LUAD cohort. (A) Lasso coefficient of prognostic ACK1-related autophagy genes. (B) Identification of the optimal risk gene signature using the LASSO model. (C) Time-dependent ROC curves.

**Additional file 2: Fig. S2.** Verification of the ACK1-related gene signature in the invalidation dataset. A total of 495 patients with LUAD were collected from three GEO datasets (GSE31210, GSE37745, and GSE50081). (A) The nomogram with a C-index of 0. 611. (B) Calibration curve.

**Additional file 3: Fig. S3.** RNA-seq revealed that differential expressed genes in the ACK1-depleted A549 cells are Enriched in the KEGG AMPK signaling pathway.

**Additional file 4: Fig. S4.** Repetition western blot results for Fig. 6. (A-E) Duplication western blot results for Fig. 6C (A), Fig. 6D (B), Fig. 6F (C), Fig. 6G (D), and Fig. 6H (E).

## Data Availability

Publicly available datasets were analyzed in this study. The TCGA datasets used in the current study can be found on the TCGA website (https://www.cancer.gov/about-nci/organization/ccg/research/structural-genomics/tcga). The datasets used and analyzed during the current study are available from the corresponding author on a reasonable request.

## Abbreviations

ACK1: Activated Cdc42-associated kinase 1
TNK2: Non-receptor tyrosine kinase 2 (TNK2)
NSCLC: Non-small cell lung cancer
LUAD: Lung adenocarcinoma
LUSC: Lung squamous cell carcinoma
AMPK: AMP-activated protein kinase
EGFR: Epidermal growth factor receptor
ALK: Anaplastic lymphoma kinase
TCGA: The Cancer Genome Atlas
GO: Gene ontology
KEGG: Kyoto encyclopedia of genes and genomes
DEGs: Differentially expressed genes
OS: Overall survival
CGP: Cancer Genome Project
IPS: Immunophenoscore
