# Supplementary figures and images for: Improvement of ACK1-targeted therapy efficacy in lung adenocarcinoma using chloroquine or bafilomycin A1
Source: Mol Med. 2023 Jan 16;29:6. doi: 10.1186/s10020-023-00602-z (PMC9843944; doi:10.1186/s10020-023-00602-z)

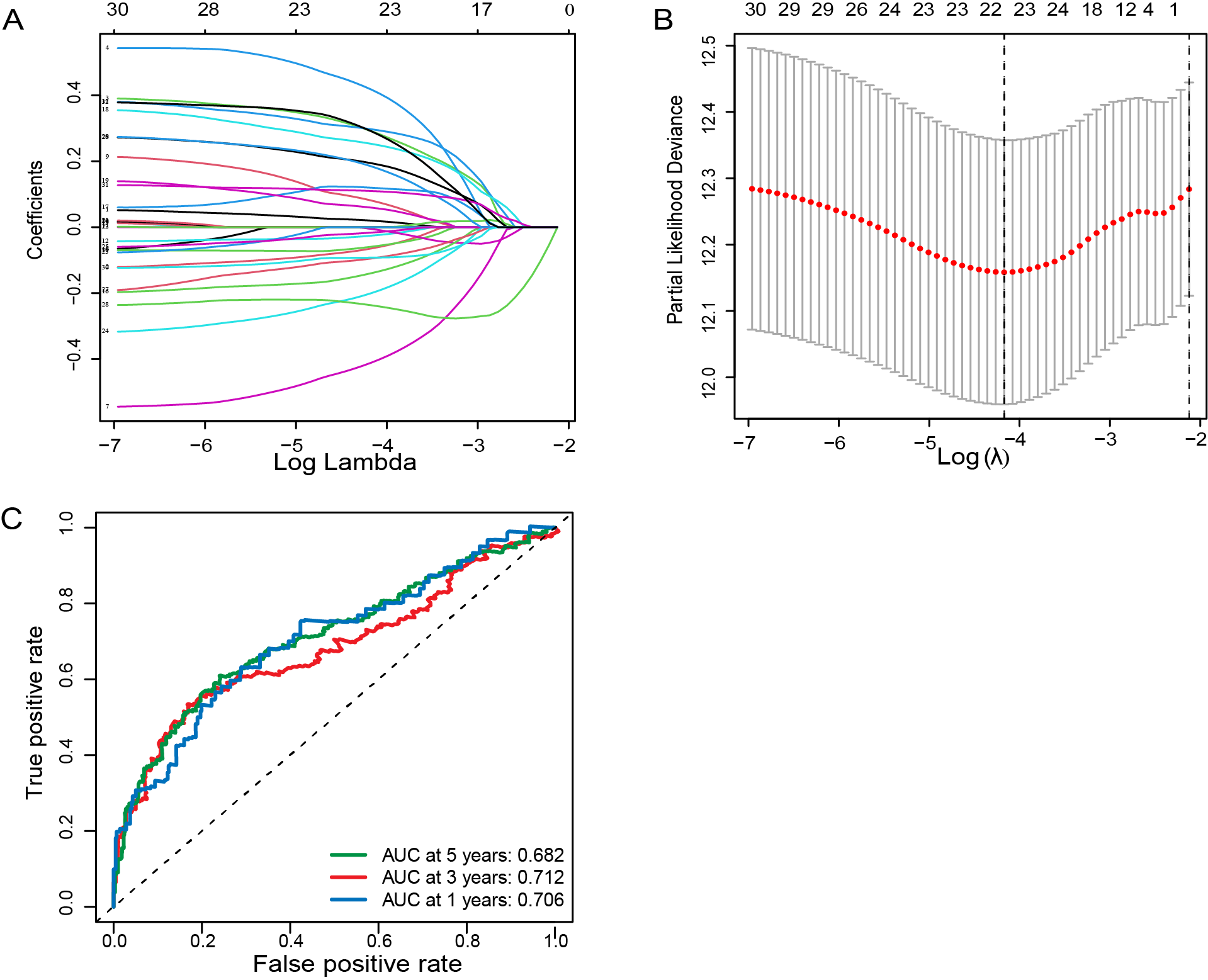

Supplement: Supplementary file 1 — Additional file 1: Fig. S1. Establishment of a prognostic signature of ACK1-correlated autophagy genes using the LASSO regression model in the TCGA-LUAD cohort. (A) Lasso coefficient of prognostic ACK1-related autophagy genes. (B) Identification of the optimal risk gene signature using the LASSO model. (C) Time-dependent ROC curves. [file 10020_2023_602_MOESM1_ESM.tif]

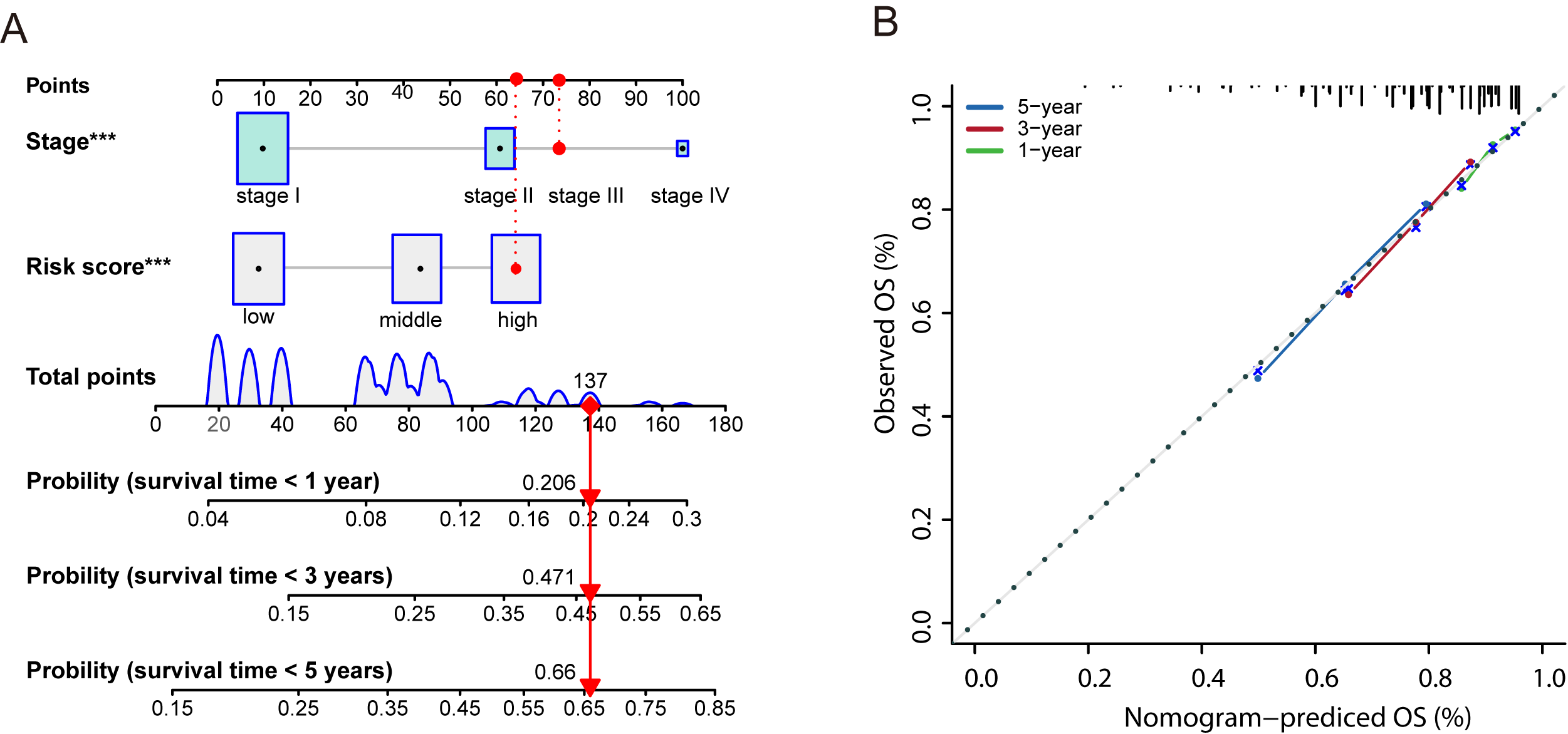

Supplement: Supplementary file 2 — Additional file 2: Fig. S2. Verification of the ACK1-related gene signature in the invalidation dataset. A total of 495 patients with LUAD were collected from three GEO datasets (GSE31210, GSE37745, and GSE50081). (A) The nomogram with a C-index of 0. 611. (B) Calibration curve. [file 10020_2023_602_MOESM2_ESM.tif]

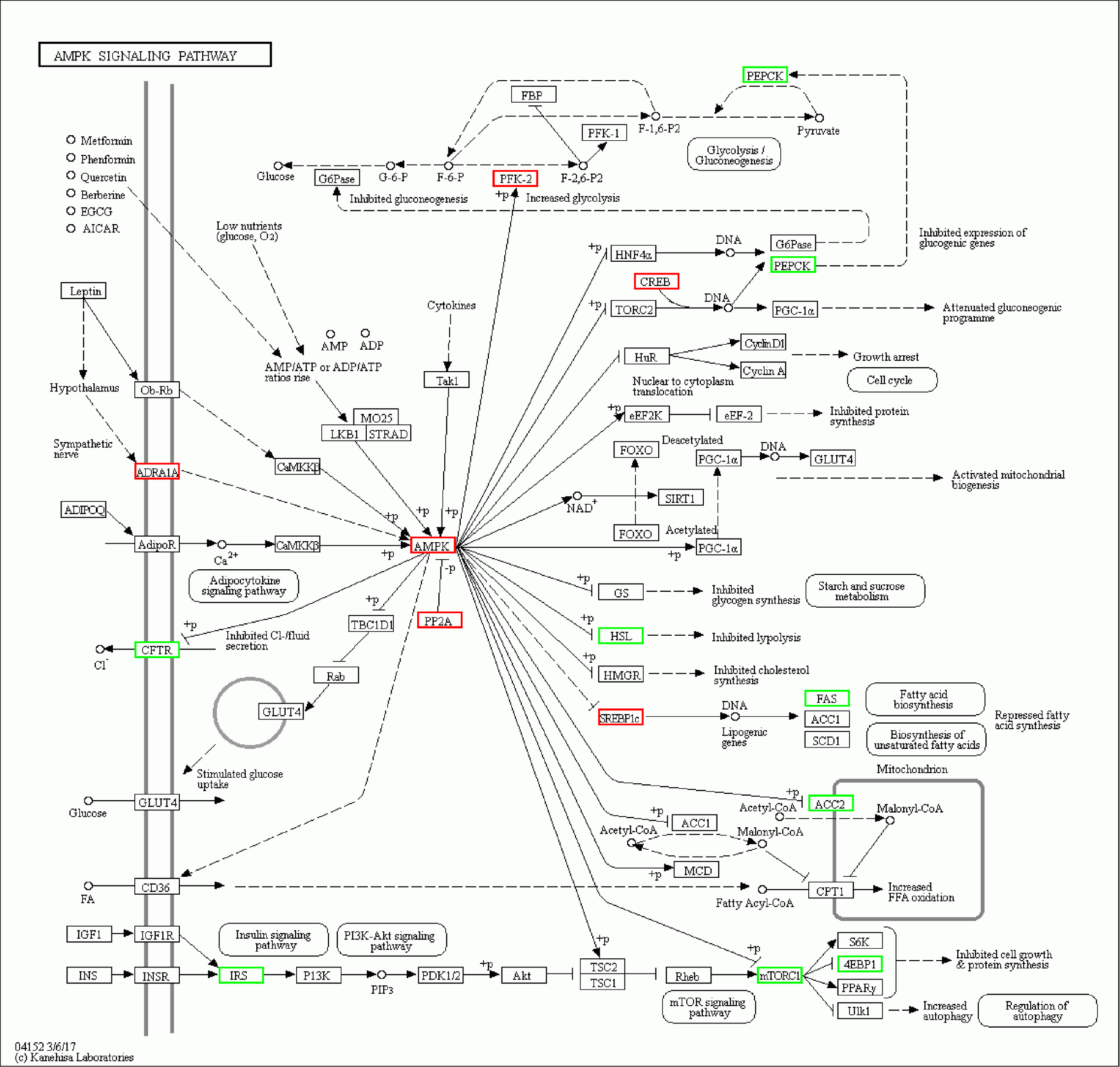

Supplement: Supplementary file 3 — Additional file 3: Fig. S3. RNA-seq revealed that differential expressed genes in the ACK1-depleted A549 cells are Enriched in the KEGG AMPK signaling pathway. [file 10020_2023_602_MOESM3_ESM.tif]

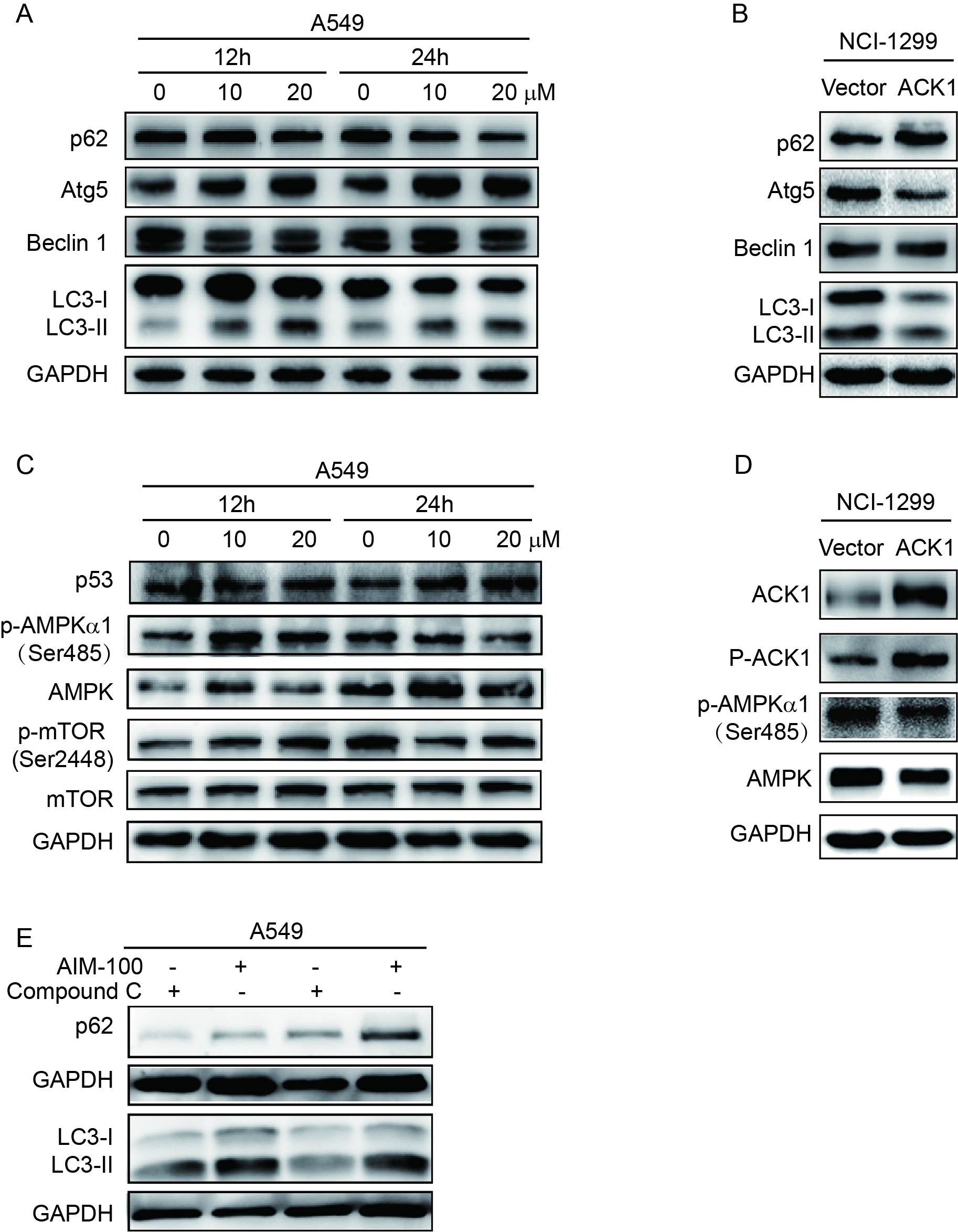

Supplement: Supplementary file 4 — Additional file 4: Fig. S4. Repetition western blot results for Fig. 6. (A-E) Duplication western blot results for Fig. 6C (A), Fig. 6D (B), Fig. 6F (C), Fig. 6G (D), and Fig. 6H (E). [file 10020_2023_602_MOESM4_ESM.tif]
